# Supplementary material for: O-GlcNAc modifications regulate lamin A tail processing
Source: bioRxiv. 2025 Mar 13:2025.03.11.642699. Preprint. [Version 1] doi: 10.1101/2025.03.11.642699 (PMC11952380; doi:10.1101/2025.03.11.642699)
Supplement: Supplement 1 [file media-1.pdf]

Figure S1

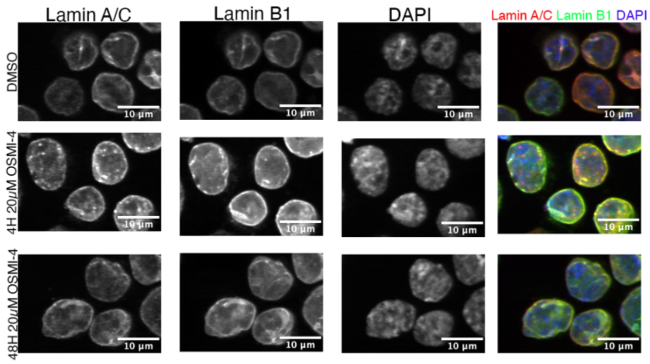

Lamin A localization after OSMI-4 treatment

Immunofluorescence staining of lamin A/C, lamin B1, and DAPI on XX mESCs treated with 20μM OSMI-4 for 4 or 48 hours or with DMSO for 48 hours. Scale bar is 10μm. Lamin A/C and lamin B1 co-localize at the nuclear periphery.

Figure S2

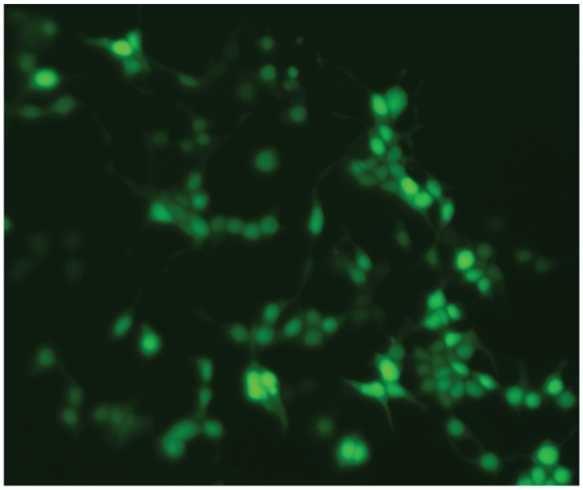

GFP-NLS-LMNA Tail (WT)

Distribution of overexpressed lamin A tail in HEK 293T cells

293T cells containing the WT lamin A tail construct were induced with 1μM Dox for 24hrs and GFP fluorescence was imaged on an Echo Revolve fluorescent microscope. Distribution of lamin A Tail peptide is primarily in the nucleus, with some lamin A tail also in the cytoplasm.
