# Supplementary material for: O-GlcNAc modifications regulate lamin A tail processing
Source: bioRxiv. 2025 Mar 13:2025.03.11.642699. Preprint. [Version 1] doi: 10.1101/2025.03.11.642699 (PMC11952380; doi:10.1101/2025.03.11.642699)
Supplement: Supplement 4 [file media-4.pdf]

Supplementary Methods Table 1

Primary Antibodies

Antigen	Manufacturer	Cat. #	Dilution IF	Dilution WB
lamin A/C	Santa Cruz	sc-376248	1:100	1:1000
O-GlcNAc	Abcam	ab2739	1:100	1:1000
OGT	Cell Signaling Technology	D1D8Q	1:100	1:1000
GFP	Proteintech	pabg1	NA	1:1000
α-Tubulin	Sigma	T5168	NA	1:2000
GAPDH	Genetex	GTX637966	NA	1:5000

Supplementary Methods Table 2

Secondary Antibodies

Antibody	Manufacturer	Cat. #	Dilution
568 AlexaFluor Goat anti Rabbit 2°	LifeTechnologies	A11011	1:1000
488 AlexaFluor Goat anti Mouse 2°	LifeTechnologies	A11029	1:1000
HRP-Conjugated Goat anti Rabit 2°	VWR	RL611-1302	1:5000
HRP-Conjugated Goat anti Mouse 2°	VWR	RL610-1302	1:5000
Anti Rabbit 2° (DyLight680 Conjugate)	Cell Signaling Technology	5366S	1:5,000 – 1:20,000
IRDye 800CW Goat anti Mouse 2°	LiCor	926-32210	1:5,000 – 1:20,000

Supplementary Methods Table 3

Table of variable modifications used in the SiLAC mass spectrometry analysis

Acetyl (Protein N-term)	HexNAc4Hex6 (N) - Rare - Motif 0 N[^P][ST]
Acetyl+Oxidation (Protein N-term M)	HexNAc4Hex6Fuc (N) - Rare - Motif 0 N[^P][ST]
Gln->pyro-Glu (N-term Q)	HexNAc4Hex6Fuc2 (N) - Rare - Motif 0 N[^P][ST]
HexNAc (N) - Rare - Motif 0 N[^P][ST]	HexNAc4Hex6SA (N) - Rare - Motif 0 N[^P][ST]
HexNAc (ST)	HexNAc4Hex6SAOx2 (N) - Rare - Motif 0 N[^P][ST]
HexNAc2 (N) - Rare - Motif 0 N[^P][ST]	HexNAc4Hex7 (N) - Rare - Motif 0 N[^P][ST]
HexNAc2Fuc (N) - Rare - Motif 0 N[^P][ST]	HexNAc5Hex3 (N) - Rare - Motif 0 N[^P][ST]
HexNAc2Hex (N) - Rare - Motif 0 N[^P][ST]	HexNAc5Hex3Fuc (N) - Rare - Motif 0 N[^P][ST]
HexNAc2Hex (ST) - Rare	HexNAc5Hex3FucSA (N) - Rare - Motif 0 N[^P][ST]
HexNAc2Hex10 (N) - Rare - Motif 0 N[^P][ST]	HexNAc5Hex4 (N) - Rare - Motif 0 N[^P][ST]
HexNAc2Hex2 (N) - Rare - Motif 0 N[^P][ST]	HexNAc5Hex4Fuc (N) - Rare - Motif 0 N[^P][ST]
HexNAc2Hex2 (ST) - Rare	HexNAc5Hex4Fuc2 (N) - Rare - Motif 0 N[^P][ST]
HexNAc2Hex2Fuc (N) - Rare - Motif 0 N[^P][ST]	HexNAc5Hex4FucSA2 (N) - Rare - Motif 0 N[^P][ST]
HexNAc2Hex3 (N) - Rare - Motif 0 N[^P][ST]	HexNAc5Hex4NeuAc (N) - Rare - Motif 0 N[^P][ST]
HexNAc2Hex3Fuc (N) - Rare - Motif 0 N[^P][ST]	HexNAc5Hex4SA (N) - Rare - Motif 0 N[^P][ST]
HexNAc2Hex4 (N) - Rare - Motif 0 N[^P][ST]	HexNAc5Hex5 (N) - Rare - Motif 0 N[^P][ST]
HexNAc2Hex4Fuc (N) - Rare - Motif 0 N[^P][ST]	HexNAc5Hex5Fuc (N) - Rare - Motif 0 N[^P][ST]
HexNAc2Hex5 (N) - Rare - Motif 0 N[^P][ST]	HexNAc5Hex5FucSA (N) - Rare - Motif 0 N[^P][ST]
HexNAc2Hex5Fuc (N) - Rare - Motif 0 N[^P][ST]	HexNAc5Hex5FucSA2 (N) - Rare - Motif 0 N[^P][ST]
HexNAc2Hex6 (N) - Rare - Motif 0 N[^P][ST]	HexNAc5Hex5SA (N) - Rare - Motif 0 N[^P][ST]
HexNAc2Hex6Fuc (N) - Rare - Motif 0 N[^P][ST]	HexNAc5Hex5SA2 (N) - Rare - Motif 0 N[^P][ST]
HexNAc2Hex7 (N) - Rare - Motif 0 N[^P][ST]	HexNAc5Hex6 (N) - Rare - Motif 0 N[^P][ST]
HexNAc2Hex8 (N) - Rare - Motif 0 N[^P][ST]	HexNAc5Hex6Fuc (N) - Rare - Motif 0 N[^P][ST]
HexNAc2Hex9 (N) - Rare - Motif 0 N[^P][ST]	HexNAc5Hex6FucSA (N) - Rare - Motif 0 N[^P][ST]
HexNAc2HexFuc (N) - Rare - Motif 0 N[^P][ST]	HexNAc5Hex6FucSA2 (N) - Rare - Motif 0 N[^P][ST]
HexNAc3Hex3 (N) - Rare - Motif 0 N[^P][ST]	HexNAc5Hex6SA (N) - Rare - Motif 0 N[^P][ST]
HexNAc3Hex3Fuc (N) - Rare - Motif 0 N[^P][ST]	HexNAc5Hex6SA2 (N) - Rare - Motif 0 N[^P][ST]
HexNAc3Hex4 (N) - Rare - Motif 0 N[^P][ST]	HexNAc5Hex6SA3 (N) - Rare - Motif 0 N[^P][ST]
HexNAc3Hex4SA (N) - Rare - Motif 0 N[^P][ST]	HexNAc6Hex7FucSA (N) - Rare - Motif 0 N[^P][ST]
HexNAc3Hex5 (N) - Rare - Motif 0 N[^P][ST]	HexNAc6Hex7SA (N) - Rare - Motif 0 N[^P][ST]
HexNAc3Hex5Fuc (N) - Rare - Motif 0 N[^P][ST]	HexNAc6Hex7SA2 (N) - Rare - Motif 0 N[^P][ST]
HexNAc3Hex5SA (N) - Rare - Motif 0 N[^P][ST]	HexNAc7Hex6SA2 (N) - Rare - Motif 0 N[^P][ST]
HexNAc3Hex5SAOxSAOxAc (N) - Rare - Motif 0 N[^P][ST]	HexNAc7Hex6SA3 (N) - Rare - Motif 0 N[^P][ST]
HexNAc3Hex6 (N) - Rare - Motif 0 N[^P][ST]	HexNAcFuc (N) - Rare - Motif 0 N[^P][ST]
HexNAc3Hex6Fuc (N) - Rare - Motif 0 N[^P][ST]	HexNAcFuc (ST) - Rare
HexNAc3Hex6SA (N) - Rare - Motif 0 N[^P][ST]	HexNAcHex (ST) - Rare
HexNAc3Hex6SA2 (N) - Rare - Motif 0 N[^P][ST]	HexNAcHexFuc (ST) - Rare
HexNAc3Hex7 (N) - Rare - Motif 0 N[^P][ST]	HexNAcHexSA (ST) - Rare
HexNAc3Hex7Fuc (N) - Rare - Motif 0 N[^P][ST]	HexNAcHexSA2 (ST) - Rare
HexNAc4Hex3 (N) - Rare - Motif 0 N[^P][ST]	HexNAcHexSAAc (ST) - Rare
HexNAc4Hex3Fuc (N) - Rare - Motif 0 N[^P][ST]	HexNAcHexSAAc2 (ST) - Rare
HexNAc4Hex4 (N) - Rare - Motif 0 N[^P][ST]	HexNAcHexSAAcSAOxAc (ST) - Rare
HexNAc4Hex4Fuc (N) - Rare - Motif 0 N[^P][ST]	HexNAcHexSAOx (ST) - Rare
HexNAc4Hex4Fuc2 (N) - Rare - Motif 0 N[^P][ST]	HexNAcHexSAOx2 (ST) - Rare
HexNAc4Hex4FucSA (N) - Rare - Motif 0 N[^P][ST]	HexNAcHexSAOxAc2 (ST) - Rare
HexNAc4Hex4SA (N) - Rare - Motif 0 N[^P][ST]	HexNAcHexSAOxSAOxAc (ST) - Rare
HexNAc4Hex5 (N) - Rare - Motif 0 N[^P][ST]	HexNAcHexSASAAc (ST) - Rare
HexNAc4Hex5Fuc (N) - Rare - Motif 0 N[^P][ST]	HexNAcHexSASAOx (ST) - Rare
HexNAc4Hex5Fuc2 (N) - Rare - Motif 0 N[^P][ST]	HexNAcHexSASAOxAc (ST) - Rare
HexNAc4Hex5FucSA (N) - Rare - Motif 0 N[^P][ST]	HexNAcSA (ST) - Rare
HexNAc4Hex5FucSA2 (N) - Rare - Motif 0 N[^P][ST]	HexNAcSAOx (ST) - Rare
HexNAc4Hex5FucSAOx2 (N) - Rare - Motif 0 N[^P][ST]	Label:13C(6) (R) - Label 1
HexNAc4Hex5SA (N) - Rare - Motif 0 N[^P][ST]	Label:13C(6)15N(2) (K) - Label 1
HexNAc4Hex5SA2 (N) - Rare - Motif 0 N[^P][ST]	Met-loss (Protein N-term M)
HexNAc4Hex5SAOx (N) - Rare - Motif 0 N[^P][ST]	Met-loss+Acetyl (Protein N-term M)
HexNAc4Hex5SAOx2 (N) - Rare - Motif 0 N[^P][ST]	Oxidation (M)
HexNAc4Hex5SAOx3 (N) - Rare - Motif 0 N[^P][ST]	Pyro-carbamidomethyl (N-term C)
HexNAc4Hex5SAOxSAOxAc (N) - Rare - Motif 0 N[^P][ST]	

Supplemental Methods Figure 4

gBlock (GFP-NLS-lamin A Tail) for HiFi Assembly of XLone-GFP-lamin A Tail:

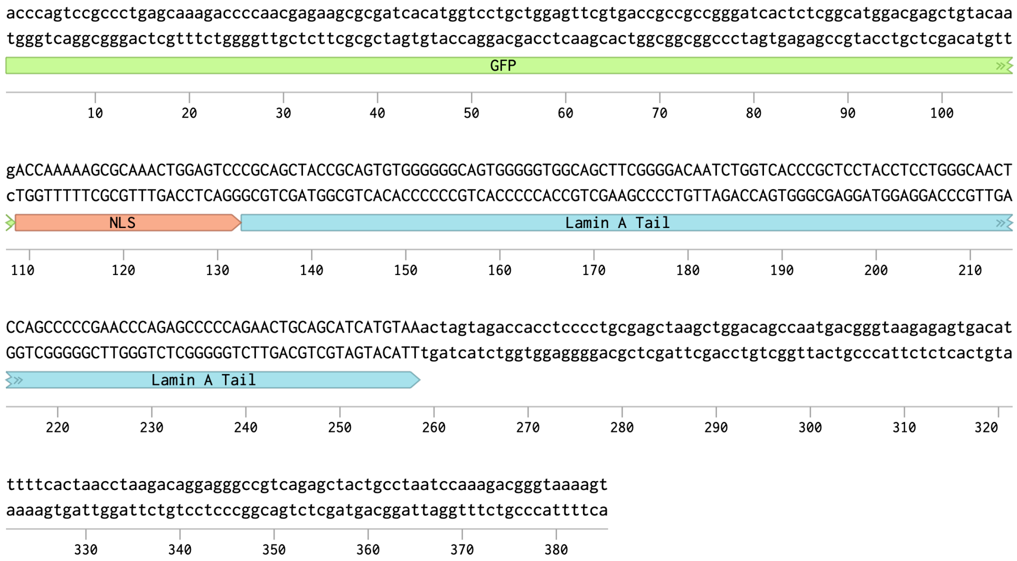

Supplementary Methods Table 5

Primers for lamin A tail construct and site directed mutagenesis

**Cell Line**	**Forward**	**Reverse**
WT lamin A Tail HiFi Assembly	CGAGCTGTACAAGACCAAA<br>AAGCGCAAACTGGAGTCCC<br>GCAGCTACCGCAGTGTGG	TGCGGTAGCTGCGGGA<br>CTCCAGTTTGCGCTTTTTG<br>GTCTTGTACAGCTCGTCCATGCCGA
Mature lamin A Tail	TAAACTAGTAGACCACCTCCC	GTAGGAGCGGGTGACCAG
L647R lamin A Tail	CCGCTCCTACCGACTGGGCAACT	GTGACCAGATTGTCCCCG
T643A lamin A Tail	CAATCTGGTCGCCCGCTCCTACC	TCCCCGAAGCTGCCACCC
S645A lamin A Tail	GGTCACCCGCGCCTACCTCCTGG	AGATTGTCCCCGAAGCTGCC
T643A+S645A lamin A Tail	CAATCTGGTCGCCCGCGCCTACC	TCCCCGAAGCTGCCACCC
D639A lamin A Tail	CAGCTTCGGGGCCAATCTGGTCACCC	CCACCCCCACTGCCCCCC
D639A+S645A lamin A Tail	GGTCACCCGCGCCTACCTCCTGG	AGATTGGCCCCGAAGCTGC
Triple Mutant lamin A Tail	CAATCTGGTCGCCCGCGCCTACC	GCCCCGAAGCTGCCACCC
